# Fatal Distant Venous Epidural Hematoma Following Posterior Cranial Fossa Surgery in a Twelve-Year old Child

**DOI:** 10.7759/cureus.12686

**Published:** 2021-01-13

**Authors:** Andrea Ortiz-Ordonez, Geovanny Jiménez-Deleg, Saif Salman

**Affiliations:** 1 Neurological Surgery, Universidad San Francisco de Quito, Colegio Ciencias de la Salud, calle Diego de Robles s/n y Pampite, 170901, Quito, ECU; 2 Neurological Surgery, Universidad San Francisco de Quito USFQ, Colegio Ciencias de la Salud, calle Diego de Robles s/n y Pampite, 170901, Quito, ECU; 3 Neurological Surgery, The Hashemite University Faculty of Medicine, Al Zarqa, JOR

**Keywords:** cranial distant epidural hematoma, hydrocephalus, postoperative complication, posterior cranial fossa tumor

## Abstract

Cranial epidural hematoma is a serious event requiring immediate intervention. This can be due to sudden traction tearing the vessels between the dura and the skull. During posterior fossa surgery, brain collapse may emerge due to the sudden reduction of prolonged elevated intracranial pressure; it could cause dura-skull detachment to create epidural hematoma even far from the surgical site. Hence, we should be aware of this complication when approaching posterior fossa tumors as it frequently leads to severe neurologic impairment or death. Here, we report a 12-year old previously healthy child who was admitted with a 4-month history of severe headache, vomiting, and right eye blindness due to increased intracranial pressure. A brain Computed Tomography (CT) scan showed obstructive hydrocephalus, and contrast-enhanced Magnetic Resonance Imaging (MRI) confirmed intraventricular posterior fossa tumor. After tumor resection, the patient developed an epidural hematoma far from the surgery site. Removal of the hematoma exposed lacerations of superior sagittal sinus due to dural detachment. Failure to control intracranial pressure resulted in a fatal outcome.

## Introduction

Cranial epidural hematoma (EDH) is blood collection in the space between the dura’s outer layer and the skull’s inner table [1]. Bleeding causes can be traumatic or non-traumatic. Infections, impaired coagulation, vascular malformations, and tumors are important non-traumatic causes [2]. This can be due to arterial injury from the branches of the Middle Meningeal Artery or venous lacerations in the Dural Venous Sinus. Herein, we discuss a rare case of distant venous EDH after posterior fossa tumor resection in a child with elevated intracranial pressure (ICP).

## Case presentation

**Figure 1:**
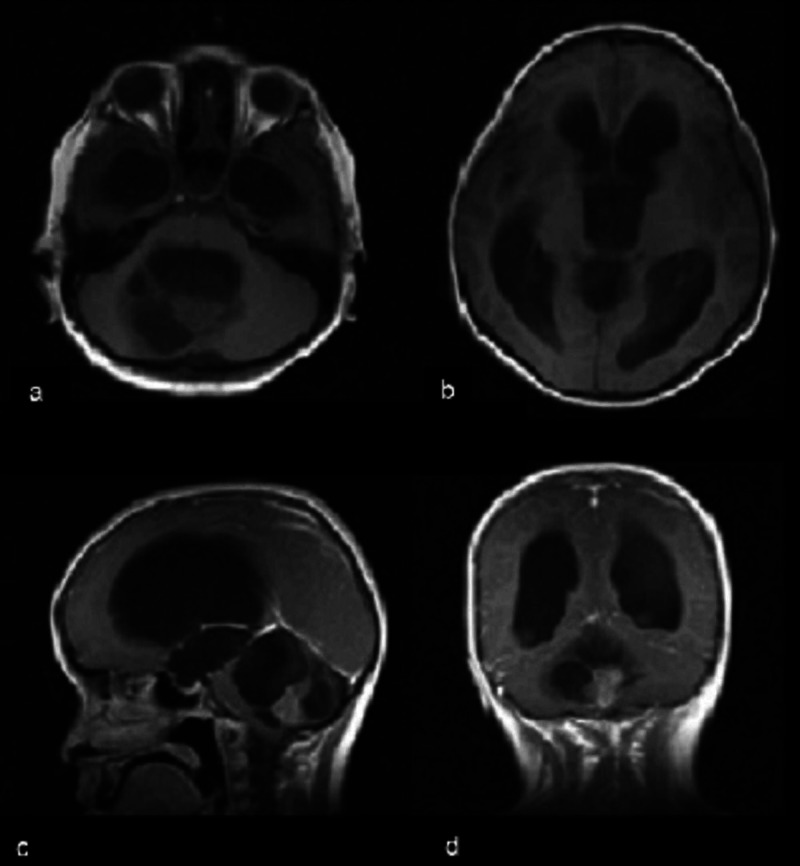
Pre-operative brain MRI A, B: axial non-contrast T1 weighted image demonstrating an isointense lesion protruding into the fourth ventricle causing obstructive hydrocephalus. C: Contrast-enhanced T1-weighted MRI, sagittal view. D: Contrast-enhanced T1-weighted MRI, coronal view.

A 12-year-old previously healthy child presented with a 4-month history of severe headache, vomiting and blurred vision that progressed to right eye blindness. Fundoscopy reported papilledema and right optic nerve atrophy compatible with raised ICP. A brain CT scan showed obstructive hydrocephalus, and subsequent contrast-enhanced T1-weighted image revealed an intraventricular posterior fossa tumor with marked homogenous enhancing (Figure 1). Platelet count, thrombin, and prothrombin time tests were within the normal range. Craniotomy for tumor removal was performed.

Operation

We placed a right intraventricular sensor, and approximately 15 ccs of cerebrospinal fluid (CSF) was drained during the procedure. A midline sub-occipital craniotomy with the patient in sitting position revealed a well-defined pinkish lobulated mass inside the fourth ventricle. Gross total tumor resection was achieved without surgical complications or significant bleeding. The patient remained hemodynamically stable during surgery. 

Post-operative course

**Figure 2:**
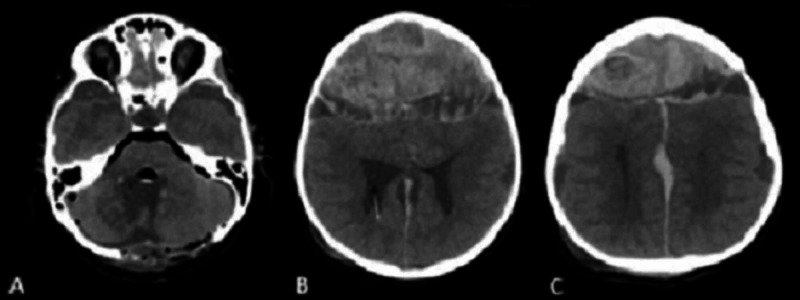
Post-operative non-contrast brain CT scan, axial view A: Gross total tumor resection and pneumocephalus. B: Acute bifrontal epidural hematoma. C: Interhemispheric hemorrhage.

The patient was managed in the Pediatric Intensive Care Unit under deep sedation due to high ICP. A control brain CT scan obtained 6 hours after surgery revealed complete removal of the posterior fossa tumor, collapsed ventricles (with right intraventricular catheter), and a large acute bifrontal epidural hematoma with interhemispheric bleeding far from the surgical site (Figure 2). External ventricular drainage remained closed since placement. No pupillary changes were seen, and emergency surgery was performed.

**Figure 3:**
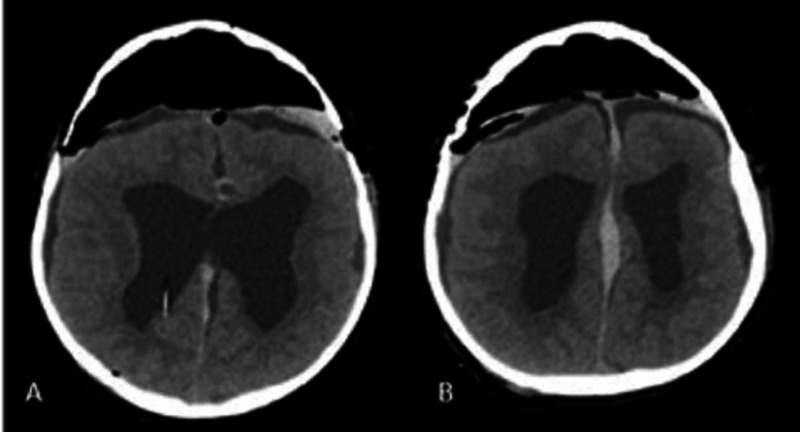
Post-operative non-contrast Brain CT scan, axial view A: Remaining frontal pneumocephalus. B: Interhemispheric hemorrhage and larger ventricles.

**Figure 4:**
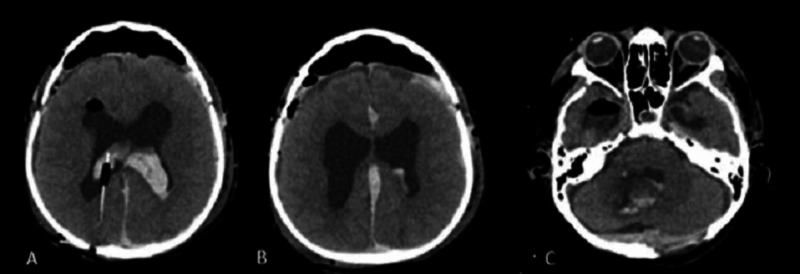
Non-contrast Brain CT scan at day 4, axial view A: Intraventricular hemorrhage. B: interhemispheric bleeding. C: Hemorrhage in the fourth ventricle.

Removal of the hematoma via bifrontal craniotomy exposed lacerations of the superior sagittal sinus due to dural detachment. A control brain CT scan obtained after 4 hours of the second surgery showed complete evacuation of the hematoma, important frontal pneumocephalus, more prominent ventricles (compared to previous imaging), and depressed brain cortex despite decompression (Figure 3). The histopathological study reported choroid plexus papilloma. Subsequent control CT-scan at 48 hours showed less frontal pneumocephalus, and intraventricular hemorrhage compromising the fourth ventricle apparently due to surgical site bleeding was found as well (Figure 4). CSF was drained in an attempt to reduce ICP without success. The patient died on day four due to refractory high ICP and hemodynamic instability. Tension pneumocephalus was suspected.

## Discussion

Cranial EDH occurring remotely after tumor resection is rare [3]. It mostly forms in the frontoparietal lobes because of the loose fixation of the dura with the skull’s inner table at that position. EDH formation peaks in the first hours postoperatively, with consequent neuro­logical deterioration and a life-threatening condition if not suspected and adequately managed [4].

The proper mechanism for the formation of epidural hematomas is not well understood. EDH may occur after cranial surgeries, near the sites of craniotomies, or burr-hole sites. However, it is not common to see an epidural hematoma occurring distantly. These hematomas can occur at many locations (on the same side or the opposite side) and many of which were reported as occurring in patients with impaired CSF drainage due to hydrocephalus [5,6].

Most authors believed that EDH occurs when the vessels between the dura and the skull are torn because of the displacement or traction forces exerted on these areas. This traction occurs after a sudden drop in ICP with CSF decompression during posterior fossa or midline tumor surgeries. It can cause brain collapse progressing the hematoma with neurologic deterioration ending with coma and/or death if not managed properly and in time. Hence, the loss of cortical support and tears in blood vessels will result in subdural, epidural, or intraparenchymal hemorrhages. Tears in cortical veins during surgical procedures in sitting position, coagulopathies, hemodynamic fluctuations (rise in blood pressure during surgery), and position-related ischemia have also been reported as possible causes [5,7,8].

Cartier-Giroux et al. in 1980 reported a case of basal ganglionic hematoma following the resection of a cerebellar metastasis. They postulated that the hemodynamic disturbances that may occur due to irritation of the medullary vasomotor center during surgery might be responsible for transient hypertension [8]. However, our patient did not develop such hemodynamic changes during surgery.

Surgeons preferred the sitting position as the approach used in our patient. It is hypothesized that the fluctuations in intracranial blood and CSF dynamics and the disruption of subcortical veins are important causes for EDH. In 1986, Seiler and Zurbrugg documented similar three cases and reviewed nine other cases performed in the sitting position [9]. Furthermore, improper neck positioning may cause ischemia, followed by reperfusion injuries that promote hematoma propagation that is similar in mechanism to an ischemic to hemorrhagic infarct conversion [7]. However, no skin or cranial bone damage due to fixation nor impaired positioning, or drastic hemodynamic changes were seen or reported in our patient during the procedure.

EDH post tumor resection

Supratentorial epidural hematoma after posterior fossa tumors resection are dreaded complications but are rarely reported [7] (Table 1).

**Table 1:** Studies reporting supratentorial bifrontal EDH after posterior fossa tumor resection in children EDH: epidural hematoma, M: male, F: female

Author, year	Age	Gender	Diagnostic	Tumor location	Hydrocephalus	Ventricular Shunt	EDH location	Outcome
Current paper	8	M	Choroid plexus papilloma	Posterior Fossa	yes	yes	supratentorial (bifrontal)	deceased
Pandey et al, 2008 [7]	5	F	Medulloblastoma	Posterior Fossa	yes	yes	supratentorial (bifrontal)	adjuvant therapy

The vessels between the dura and the skull are torn when the brain volume is massively reduced. The earliest information available about such a complication is that published by Tsugane et al. in 1976 when they suggested that a decrease in ventricular pressure by the ventricular tap during surgery may play an important role in the development of extradural hematoma [10]. The cortex collapse develops traction forces over the vessels (slightly adhered to the skull) and may cause lacerations as seen in our patient.

Seyithanoglu et al. suggested that in certain patients, the adhesions between the skull and dura are less important than dura-arachnoid adhesions and this would contribute to the development of a hematoma in the epidural area rather than the subdural space in cases of over-drainage [11]. Hence, the acute epidural hematoma is related to CSF drainage resulting in vessel tearing and dural detachment; this often occurs before herniation. However, in some cases, neither the duration of hydrocephalus nor the type of the tumor can determine the cause of EDH, as Jan et al. [12], and Wolfsberger et al. reported [13].

Ventriculoperitoneal shunts and ventriculostomies

EDH following ventriculostomies and ventriculoperitoneal shunt is a significant complication that could be due to excessive drainage, presenting mainly as subdural bleeding (incidence of 5-55%), where rapid decompression of hydrocephalus results in upward herniation of the infratentorial structures [10]. Furthermore, there are reports of subdural hygromas, intraventricular hemorrhages, intraparenchymal hemorrhages, and extradural hematomas because of shunt malfunction or surgical complications, with consequent neurological deterioration and death. Consequently, this mandates proper interventions, such as bleeding control, minimizing CSF spillage with ventricular catheter insertion, proper surgical techniques, use of high or medium pressure valves, slowly resuming the full upright position, and postoperative CT scan follow up [4].

The most extensive case series of EDH (42 cases) following decompression of ventricles, was done by Odake and Matsumoto [14], which includes both ventriculoperitoneal shunt and procedures for CSF drainages (ventricular puncture, ventriculography, ventriculoatrial shunts, and others). Furthermore, Kalia et al. confirmed the development of numerous extradural hematomas in a child after ventriculostomy because of heavy drainage and brain collapse due to traction on the middle meningeal artery [15]. 

Postsurgical pneumocephalus and high intracranial hypertension

Postsurgical pneumocephalus is often observed during the early postoperative period. It can be due to dural tear that allows air introduction via 2 possible ways: ball-valve mechanism from Valsalva (straining, coughing, sneezing, or mechanical ventilation) or vacuum phenomenon caused by CSF loss [16]. Tension pneumocephalus should be suspected and treated as it results in high ICP with eventually fatal outcome as in our patient. 

Management

A postoperative brain CT scan is routinely done, especially if the patient did not recover appropriately. Thus, postsurgical patient management is more efficient by maintaining a high index of suspicion and early detection of any complication [3]. If a large volume of bleeding is found, for example, immediate surgical evacuation should be performed as in our patient. Therefore, even silent EDH or those at remote sites can be diagnosed and controlled [3].

## Conclusions

Remote cranial epidural hematoma rarely occurs after surgery, but if it does, then it is a serious event that requires immediate intervention. Sudden traction on the vessels between the dura and the skull can cause them to be torn; however, the mechanism of formation is not entirely understood. It can be related to the rapid drop in ICP due to the reduction of CSF following excessive ventricular drainage or tumor excision.

It is critical to remember such complications when suspecting hydrocephalus or performing surgeries in the posterior fossa with increased ICP. Even though such cases have rarely been reported in the literature, EDH carries complications and fatal consequences. Sometimes, patient management requires emergency surgery to relieve elevated ICP approaching the cause, as rapid bleeding evacuation carries beneficial outcomes, even for post-surgical silent or remote EDH. Consequently, a postoperative CT scan must be routinely done after surgical treatment, especially if the patient did not recover directly after surgery or experienced sudden neurological deterioration. Surgeons must recognize that surgical intervention alone might not be enough to control increased ICP; hence, neurointensive care is a cornerstone.

Additionally, postsurgical pneumocephalus should not be diminished. We recommend the distinction of uncomplicated pneumocephalus from tension pneumocephalus as it is of vital importance as the latter constitutes a neurosurgical emergency that can result in fatal outcome as in our patient.

## References

[1] Rosenthal AA, Solomon RJ, Eyerly-Webb SA (2017). Traumatic epidural hematoma: patient characteristics and management. Am Surg.

[2] Tamburrelli FC, Meluzio MC, Masci G, Perna A, Burrofato A, Proietti L (2018). Etiopathogenesis of traumatic spinal epidural hematoma. Neurospine.

[3] Yu J, Yang H, Cui D, Li Y (2015). Retrospective analysis of 14 cases of remote epidural hematoma as a postoperative complication after intracranial tumor resection. World J Surg Oncol.

[4] Noleto G, Neville IS, Tavares WM, Saad F, Pinto FC, Teixeira MJ, Paiva WS (2014). Giant acute epidural hematoma after ventriculoperitoneal shunt: a case report and literature review. Int J Clin Exp Med.

[5] Avci E, Dagtekin A, Baysal Z, Karabag H (2010). Intraoperative supratentorial epidural haematoma during removal of a huge posterior fossa dermoid cyst. Neurol Neurochir Pol.

[6] Louzada PR, Requejo PR, Barroso MV, Vaitsman RP, Machado AL, Paiva MS, Salame JM (2012). Bilateral extradural haematoma after acute ventricular over-drainage. Brain Inj.

[7] Pandey P, Madhugiri VS, Sattur MG, Devi BI (2008). Remote supratentorial extradural hematoma following posterior fossa surgery. Child’s Nerv Syst.

[8] Cartier-Giroux J, Mohr G, Sautreaux JL (1980). Supratentorial hemorrhage of hypertensive origin during operation: an unusual complication of surgery on the posterior fossa in the sitting position (author’s translation). Neurochirurgie.

[9] Seiler RW, Zurbrügg HR (1986). Supratentorial intracerebral hemorrhage after posterior fossa operation. Neurosurgery.

[10] Tsugane R, Sugita K, Sato O (1976). Supratentorial extradural hematomas following posterior fossa craniectomy (author's translation). No Shinkei Geka.

[11] Seyithanoglu H, Karagoz Guzey F, Emel E, Ozkan N, Aycan A (2009). Chronic ossified epidural hematoma after ventriculoperitoneal shunt insertion. a case report. Turk Neurosurg.

[12] Jan M, Gouaze A, Elie A, Lapierre F, Santini JJ (1977). Extra dural hematoma complicating ventricular decompression during posterior fossa exploration (author's translation). Neurochirur.

[13] Wolfsberger S, Gruber A, Czech T (2004). Multiple supratentorial epidural haematomas after posterior fossa surgery. Neurosurg Rev.

[14] Odake G, Matsumoto S (1981). Supratentorial epidural hematoma as a complication of internal decompression. Neurol Med Chir.

[15] Kalia KK, Swift DM, Pang D (1993). Multiple epidural hematomas following ventriculoperitoneal shunt. Pediatr Neurosurg.

[16] Pulickal GG, Sitoh YY, Ng WH (2014). Tension pneumocephalus. Singapore Med J.

